# Research on the biological mechanism and potential application of CEMIP

**DOI:** 10.3389/fimmu.2023.1222425

**Published:** 2023-08-18

**Authors:** Yang Liu, Gang Hu, Yuetong Li, Xinyi Kong, Kaming Yang, Zhenlin Li, Wanwen Lao, Jiaxin Li, Jianhua Zhong, Shitong Zhang, Yuxin Leng, Changlong Bi, Aixia Zhai

**Affiliations:** ^1^ Department of Laboratory Medicine, The Eighth Affiliated Hospital, Sun Yat-sen University, Shenzhen, China; ^2^ The Eighth Affiliated Hospital, Sun Yat-sen University, Shenzhen, China; ^3^ Department of Endocrinology, The Eighth Affiliated Hospital, Sun Yat-sen University, Shenzhen, China; ^4^ Department of General Practice, The Eighth Affiliated Hospital, Sun Yat-sen University, Shenzhen, China; ^5^ Department of Critical Care Medicine, Peking University Third Hospital, Beijing, China

**Keywords:** CEMIP, HA, pro-inflammatory factor, tumor, cell microenvironment, fibrosis

## Abstract

Cell migration–inducing protein (CEMIP), also known as KIAA1199 and hyaluronan-binding protein involved in hyaluronan depolymerization, is a new member of the hyaluronidase family that degrades hyaluronic acid (HA) and remodels the extracellular matrix. In recent years, some studies have reported that CEMIP can promote the proliferation, invasion, and adhesion of various tumor cells and can play an important role in bacterial infection and arthritis. This review focuses on the pathological mechanism of CEMIP in a variety of diseases and expounds the function of CEMIP from the aspects of inhibiting cell apoptosis, promoting HA degradation, inducing inflammatory responses and related phosphorylation, adjusting cellular microenvironment, and regulating tissue fibrosis. The diagnosis and treatment strategies targeting CEMIP are also summarized. The various functions of CEMIP show its great potential application value.

## 1 Background

**Figure 1:**
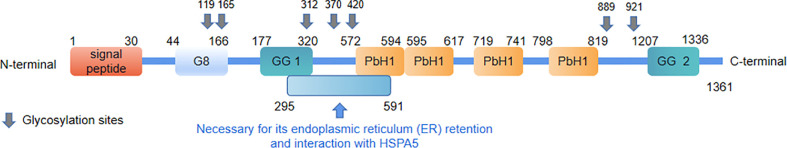
The protein structure of CEMIP. The numbers refer to the amino acid positions that flank the different domains.

In 1999, Nagase et al. (1) first reported the gene cell migration–inducing protein (CEMIP) with unknown functions. Four years later, Abe et al. (2) analyzed the genes of patients with hereditary deafness using microarray technology by constructing an inner ear complementary DNA (cDNA) library and, for the first time, confirmed that mutations of the *CEMIP* gene locus may be the cause of the disease. The coding gene of *CEMIP* is located on chromosome 15q25.1 and encodes a 153-kDa protein containing 1,361 amino acids (2, 3). Because of the difficulty of purifying the protein, its atomic structure has not yet been fully resolved. However, with the joint efforts of Birkenkamp-Demtroder et al. and Yoshida et al., we have gained some information of the protein structure about the CEMIP (4, 5). CEMIP contains a signal peptide composed of 30 amino acids, one G8 domain, two GG domains, four PbH1 repeats, and seven glycosylation sites (4, 5). In addition, it has a key sequence that keeps it in the endoplasmic reticulum (ER) and interacts with heat shock protein A5 (HSPA5) (**Figure 1**) (6, 7).

CEMIP, also known as KIAA1199 and hyaluronan-binding protein involved in hyaluronan depolymerization (HYBID), was first found to be expressed in the cochlea and, subsequently, in other human organs, particularly in the skin and cartilage, and secretory CEMIP was also detected in serum (2). At the subcellular level, CEMIP was found to be accumulated in the ER, which was confirmed by electron microscopy (8). In 2013, Evensen et al. (6) found that the secretion of CEMIP needs to pass through the ER, which forms a stable complex with binding immunoglobulin protein (BiP). They identified a novel sequence in CEMIP that is necessary for its ER localization, BiP interaction, and enhanced cell migration.

Studies involving CEMIP have focused on the field of oncology, which plays an important role in promoting proliferation and metastasis in a variety of tumors. Abnormal expression of CEMIP can also regulate non-neoplastic diseases, such as inflammation and infection. Studies have shown that the anti-CEMIP monoclonal antibody (anti-CEMIP), ipriflavone, and paclitaxel (PTX) combined with the latest nanosphere-targeted drug delivery technology can make CEMIP as a therapeutic target to inhibit inflammation or tumor development (9, 10).

Summarizing the pathophysiological mechanism of CEMIP in diseases has important guiding significance for exploring the diagnosis and treatment strategies targeting CEMIP. The critical role of CEMIP in promoting tumor migration and invasion has been identified by numerous studies, but the precise molecular mechanism is still unknown. Research on CEMIP in non-neoplastic diseases is increasing, including infection, fibrosis, and activation of immune cells, especially in inflammatory osteoarthritis (OA). This suggests that CEMIP may have more biological functions. Therefore, we reviewed the regulatory mechanisms and biological activities of CEMIP to understand its current research progress. In addition, on the basis of our current understanding of CEMIP, we look forward to its potential applications and future research focus.

## 2 Regulation of CEMIP expression

**Figure 2:**
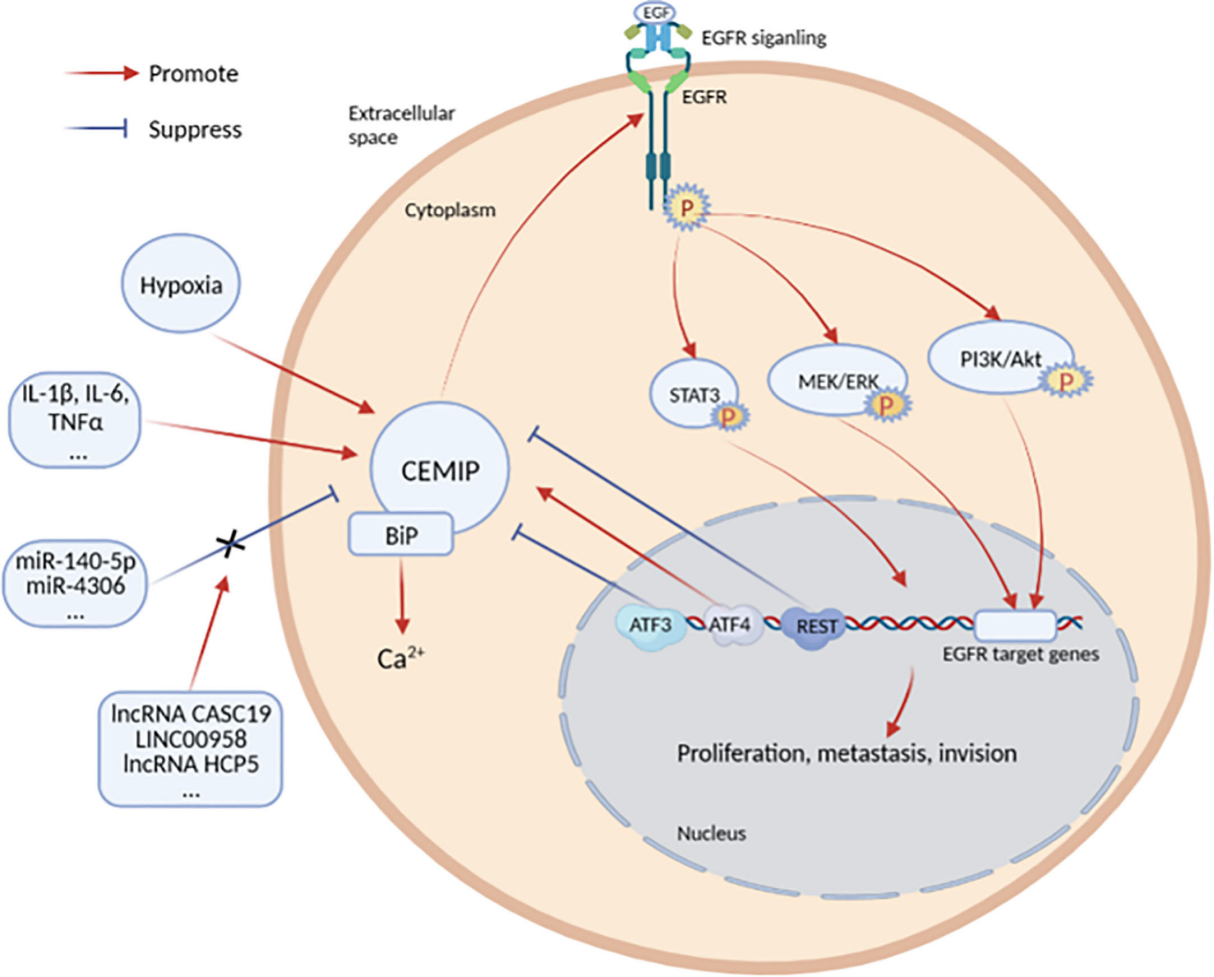
Cytokines and signaling pathways regulating the expression of CEMIP.

The expression of CEMIP is regulated by inflammatory factors, transcription factors, noncoding RNAs, and histone methylation. However, there are also some disputes in these studies (**Figure 2**).

### 2.1 Inflammatory factors

Inflammation is an important immune defense mechanism in the body. The pro- and anti-inflammatory factors produced in this defense process work together to maintain the homeostasis of the cellular microenvironment. Interleukin-1β (IL-1β), IL-6, tumor necrosis factor–alpha (TNF-α), and transforming growth factor–beta (TGF-β) are involved in regulating the activity of CEMIP.

CEMIP participates and plays an important role in the process of IL-1β–mediated pro-inflammatory. Ohtsuki et al. (11) used IL-1β, TNF-α, IL-6, and IL-8 to stimulate chondrosarcoma cells (OUMS-27) for 6 h, and the results showed that CEMIP expression was significantly increased and peaked at 12 h. The expression of CEMIP may be induced by IL-1β/IL-1R binding to activate extracellular signal-regulated kinase (ERK) phosphorylation and to enhance nuclear translocation of nuclear factor kappa B (NF-κB). Another study showed that the IL-1β–induced Wnt/β-catenin pathway activation was inhibited by KIAA1199 silencing, but the molecular mechanisms of CEMIP on the IL-1β–induced Wnt/β-catenin pathway were unknown (12).

In OA, IL-6 stimulates chondrocytes to produce CEMIP, which increases the level of osteoarticular inflammation with increased degradation of high–molecular weight hyaluronan (HMW-HA). IL-6 activating NF-κB *via* phosphatidylinositol 3-kinase (PI3K)/protein kinase B (PKB/AKT) signaling is thought to be the main pathway for inducing CEMIP expression in rheumatoid arthritis (RA) fibroblast-like synoviocytes (FLSs) (13). Combined treatment with IL-6 or TNF-α leads to the significantly increased expression of CEMIP (14).

Shimizu et al. (15) stimulated chondrocytes with eight cytokines (TNF-α, TGF-β, IL-1α, histamine, Insulin-like growth factor-1 (IGF-1), vascular endothelial-derived growth factor (VEGF), basic fibroblast growth factor (bFGF), and prostaglandin E2 (PGE2), only TNF-α stimulated chondrocytes to express CEMIP. However, there are some controversies in these studies. For example, the highest level of CEMIP was induced by IL-1β in chondrocytes, whereas the highest level of CEMIP was induced by IL-6 and TNF-α in synovial fibroblasts (16). Stimulation of chondrosarcoma cells with cytokines IL-1β, TNF-α, IL-6, and IL-8 separately resulted in a significant increase in CEMIP, whereas treatment with a mixture of pro-inflammatory cytokines (TNF-α, IL-1β, and IL-6) in human dermal fibroblasts decreased the CEMIP messenger RNA (mRNA) levels and protein expression (17).

In early tumors, the TGF-β pathway induces apoptosis and inhibits tumor cell proliferation. Instead, by late stage, it has a tumor-promoting effect by regulating genomic instability, epithelial–stromal transition (EMT), new angiogenesis, immune escape, cell motility, and metastasis (18). Deroyer et al. (19) found that TGF-β upregulates CEMIP through the activin receptor-like kinase (Alk) 5/plasminogen activator inhibitor (PAI)-1 (Alk5/PAl-1) pathway in dedifferentiated chondrocytes and is, therefore, a pro-fibrotic mediator. Nagaoka et al. (20), however, came to the opposite conclusion, finding that CEMIP expression was reduced in skin fibroblasts after TGF-β stimulation, reducing fibrosis (20, 21).

The different phenomena mentioned above might be explained by the use of different cell lines. In addition, using primary cell lines can give a more accurate representation of the body’s inflammatory response as compared to the outcomes obtained with tumor cell lines. Although the existing evidence cannot fully explain the molecular mechanisms of the regulation of CEMIP expression by inflammatory factors, existing reports have confirmed that there is a significant correlation between them. Revealing the molecular mechanism of the regulation of CEMIP by inflammatory factors is the focus of future research.

### 2.2 Transcription factors

Transcription factors also play an essential role in regulating CEMIP (22). In a study of gastric cancer (GC), activating transcription factor 3 (ATF3) inhibited the activity of *CEMIP* promoter (23). When ATF3 is low-expressed, CEMIP expression increases, which promotes the proliferation and migration of GC cells and, conversely, inhibits the progression of GC. ATF3-short hairpin RNA/AGS implantation in nude mice accelerates tumor growth and increases the probability of lung metastasis of GC cells. As a protein that protects against oxidative stress and activates protective autophagy, ATF4 plays an important role in the regulation of CEMIP expression. From the experiments of Yu et al. (24), it was found that ATF4 has a positive regulatory effect on CEMIP, and, from the conclusion of the rescue experiments, both play a function of anti-anoikis. Dual-luciferase reporter assay also confirmed that ATF4 may directly bind to the CEMIP promoter region, thereby initiating the transcriptional regulation of CEMIP. In both studies, ATF3 and ATF4 belong to the same family and bind to the CEMIP promoter region but play opposite roles. ATF3 inhibits CEMIP promoter activity, whereas ATF4 promotes CEMIP promoter activity. They have different physiological functions. This difference may be due to the different binding sites of the two transcription factors, but the exact mechanism is still unknown.

Repressor element 1 (RE1) silencing transcription factor (REST) is a transcription factor that regulates neuronal-associated genes. In recent studies on breast cancer, it is found that REST directly binds to the RE1 site of *CEMIP* gene, inhibits CEMIP expression, and weakens the proliferative capacity of breast cancer cells (25). There were four NF-κB binding sites in the promoter region of CEMIP. The sequence of binding sites all contained a sequence with continuous bases, and the highest binding site score was a TATA box between −32 and 23 (26).

The regulatory functions of some transcription factors mentioned above provide us with new insights into the regulatory mechanism of CEMIP. Some sequences in the promoter region of CEMIP may promote or inhibit the transcription of CEMIP. Further studies are needed to reveal the regulatory mechanism of CEMIP transcription.

### 2.3 Non-coding RNA

Non-coding RNAs (ncRNAs) include micro-RNAs (miRNAs), circle RNAs (circRNAs), and long ncRNAs (lncRNAs). Studies have shown that more than half of the DNA of higher organisms is transcribed into RNA, the vast majority of which is ncRNAs. Although the mechanisms about ncRNA remain unclear, we can know that it plays an important role in tumor progression.

miRNA is a kind of ncRNAs. It can regulate the target mRNA by destroying its stability and inhibiting its translation. Studies have proved that there are more than 10 miRNAs that combine with CEMIP 3′untranslated region, such as miR-148a-3p, miR-4306, miR-296-3p, miR-4677-3p, miR-486-3p, miR-4656, miR-140-5p, miR-17-5p, miR-216a, miR-1248, miR-486-5p, miR-29c-3p, and miR-34c-5p (27). After being combined with these miRNAs, the expression level of CEMIP is downregulated that affects the progression of tumor cells. In addition, some lncRNAs and circRNAs were also found to be able to bind to these miRNAs in these studies, such as LINC00958 (28), circ_001653 (29), lncRNA CASC19 (30, 31), HCP5 (32), circ_0004585 (33), and circ-BPTF (34–36) (**Table 1**). They acted as competing endogenous RNAs (ceRNAs) to sponge miRNAs and resulted in low expression of miRNAs. Moreover, Musashi RNA-binding protein 1 (MSI 1), a specific protein, could bind to miR-34c-5p, which blocks the inhibition of miR-34c-5p on CEMIP expression (**Table 1**). After that, the expression level of CEMIP was significantly upregulated. Regulating the expression level of these ncRNAs can indirectly regulate the expression of CEMIP, thereby alleviating the progression of tumors or other diseases, which is expected to be a new treatment strategy.

**Table 1:** Summary of miRNAs that inhibit CEMIP expression and their corresponding target molecules.

miRNA	Targeting molecules	Related diseases	Reference
miR-148a-3p	NA	Gastric cancer cells	(37)
miR-4306	LINC00958	Osteosarcoma	(28)
miR-296-3p	NA	Preeclampsia	(38)
miR-4677-3p	NA	Gastric cancer	(39)
miR-486-3p	circ_001653	Intervertebral disc degeneration	(29)
miR-4656	HCP5	Prostate cancer cell	(32)
miR-140-5p	CASC19	Colorectal cancer Retinoblastoma	(30, 31)
miR-17-5p	NA	Gastric cancer	(23)
miR-216a	NA	Colorectal cancer	(40)
miR-1248	circ_0004585	PCa	(33)
miR-486-5p	circ-BPTF	Lung cancer, papillary thyroid cancer, chronic obstructive pulmonary disease	(34–36)
miR-29c-3p	NA	Gastric cancer	(41)
miR-34c-5p	MSI1	Colorectal cancer	(27)

NA, not applicable.

### 2.4 Histone methylation modifications

Histone methylation can regulate gene transcription. Hsieh et al. (44) found that increased trimethylation of lysine on histone H3 (H3K27me3) is associated with the inactivation of CEMIP, which can reduce the growth and migration of tumor cells in triple-negative breast cancer (TNBC). Hypoxia-inducible factor 2α (HIF-2α) is induced by hypoxia and bounds to the promoter of CEMIP while inhibiting the demethylation of Jarid1A, thereby increasing the effect of H3K4me3 on the CEMIP promoter and the expression of CEMIP (45).

### 2.5 Hypoxia

In the process of tumorigenesis and development, because of the rapid proliferation of solid tumor tissues and the untimely and inadequate construction of blood vessels, the overall state of hypoxia is often presented in the process of the growth of tumors (46). Another characteristic of tumor cells is that, even under aerobic conditions, they also undergo glycolysis to produce lactic acid and to make cells infiltrate in an acidic environment (47). In hypoxic and acidic environments, tumor cells and surrounding normal cells often suffer apoptosis, and the cellular fragments and chemokines produced during apoptosis cause inflammatory reactions in surrounding tissues, leading to inflammatory cell infiltration and the release of inflammatory factors (48).

The cellular response to hypoxia is generally regulated by the HIF (49). As early as 2015, studies confirmed that there is a certain correlation between hypoxia and the expression of CEMIP. Evensen et al. (45) found that CEMIP upregulation is ensured by immunohistochemistry only in the part of the cancer cells in colon cancer. HIF-2α is stably expressed under a hypoxic condition. Meanwhile, HIF-2α directly combines with the CEMIP promoter to increase the expression of CEMIP, whereas HIF-1α has no such function. However, Wang et al. (50) found that CEMIP and HIF-1α were both elevated, and there may be a certain correlation with the expression of CEMIP and HIF-1α, showing significant differences in TNM staging compared with patients with a low level of expression. This was also confirmed by Oba et al. (51), which showed that CEMIP and HIF-1α are significantly associated in cancer tissues, and *in vitro* experiments confirmed that hypoxia induces the upregulation of CEMIP and HIF-1α expression and enhances the migration ability of pancreatic ductal cancer cells.

In the hypoxic environment, CEMIP and BiP are regarded as new regulatory axes. CEMIP regulates the growth of tumor cells by regulating downstream molecular BiP to reduce cell apoptosis, activate autophagy, enhance glucose uptake, and survive in the harsh environment of hypoxia. *In vivo* experiments have shown that reducing the expression of BiP in a hypoxic environment leads to the reduction of glucose uptake by tumor cells, thus leading to tumor regression, which may become a potential therapeutic target (52).

## 3 Biological mechanism of CEMIP

**Figure 3:**
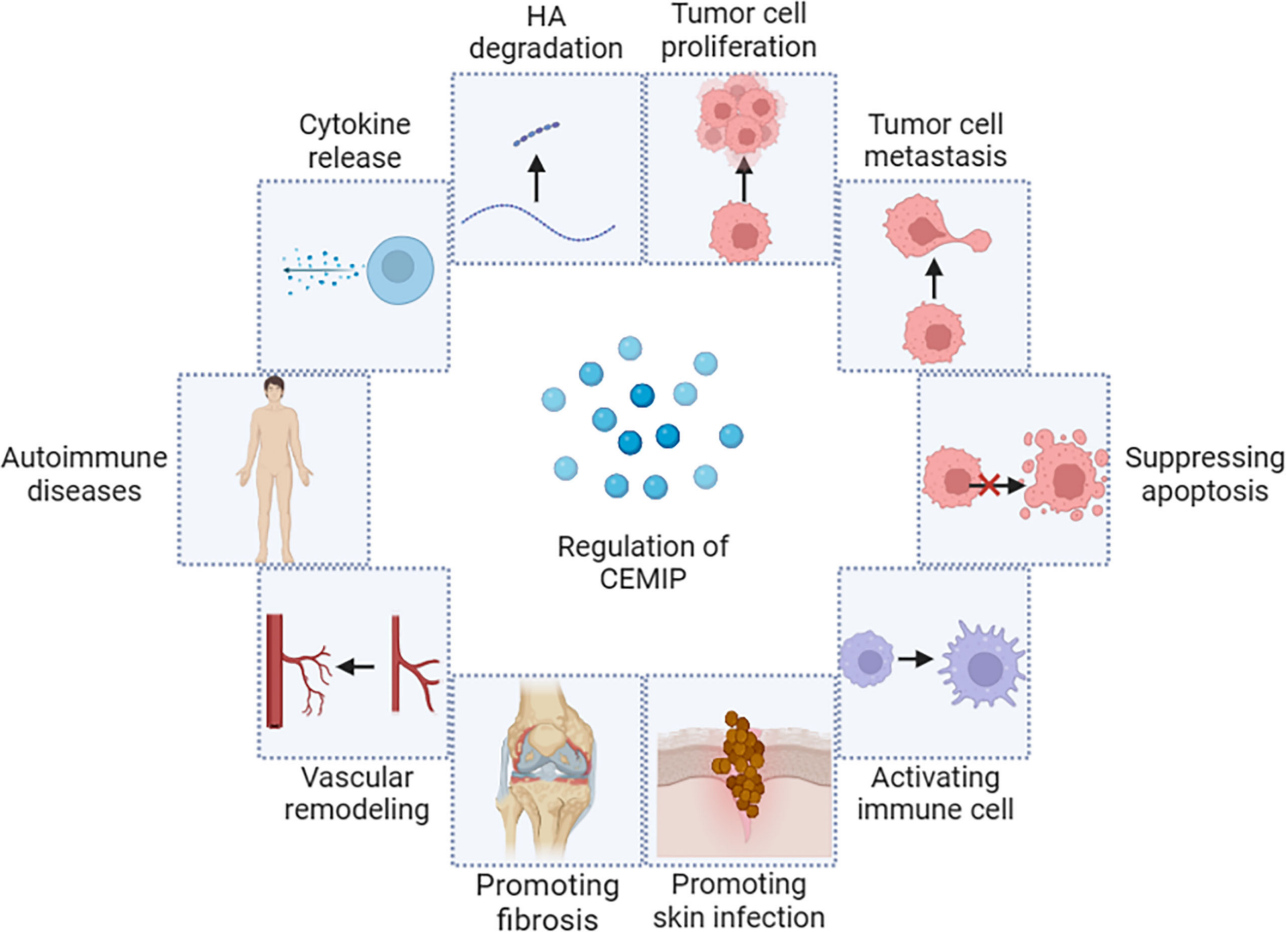
Biological mechanism of CEMIP.

The biological mechanism of CEMIP is multifaceted and complex. As a newly discovered HA enzyme, CEMIP promotes the degradation of HA, and the degradation products will induce local inflammation (21, 53–57). CEMIP affects the expression of genes related to cell cycle regulation, proliferation, migration, and apoptosis (29, 58, 59). CEMIP activates downstream regulatory molecules by promoting the phosphorylation level of some signaling molecules and further regulates target genes that relate to tumor cell activities among multiple signaling pathways, including Notch, Wnt/β-catenin, epidermal growth factor receptor (EGFR), PI3K/Akt, signal transducer and activator of transcription 3 (STAT3) (60–68). At the same time, CEMIP also plays a complex and important role in promoting fibrosis and bacterial infection (69, 70) (**Figure 3**).

### 3.1 CEMIP promoted HA degradation

HA is a high–molecular weight linear glycosaminoglycan (GAG), which is a disaccharide unit GAG composed of D-glucuronic acid and N-acetylglucosamine (71). Hyaluronic acid (HA) is ubiquitous in vertebrate tissues and a major component of the extracellular matrix (ECM), providing structural and functional integrity to cells and organs (72). Skin is the main determining organ for HA turnover, which contains about half the HA throughout the body. The metabolic half-life of HA is 1–1.5 days, and roughly one-third of HA is renewed every day. HA is rapidly depolymerized within tissues, ranging from supermolecules of 1,000–10,000 kDa to medium-sized fragments of 10–100 kDa present in the extracellular environment (73).

The molecules involved in HA metabolism are classified as hyaluronan synthase and hyaluronidase. Hyaluronan synthase includes HAS1, HAS2, and HAS3, and hyaluronidase includes HYAL-1, HYAL-2, HYAL-3, HYAL-4, PH20/SPAM1, and HYAL-P1 (74, 75). In recent years, it has been gradually identified that CEMIP and transmembrane protein 2 (TMEM2) also have the function of hyaluronidase, but their mechanisms of action are not completely clear (76–78).

The studies show that CEMIP can promote the degradation of HMW-HA to low–molecular weight hyaluronan (LMW-HA). LMW-HA, formed by the degradation of CEMIP, is often detected in some diseases (13, 16, 79, 80). However, the molecular mechanism of CEMIP in the degradation of HA is not clear. The protein structure of CEMIP does not have a domain similar to that of the known HA enzyme, and it lacks HA-link modules (5, 76). Although CEMIP is necessary in the process of HA decomposition, it does not detect any HA enzyme activity in the absence of cells *in vitro*. These results indicate that living cells are essential for CEMIP-mediated HA degradation.

CEMIP plays an important role in normal osteogenic development. CEMIP counteracts this inhibition by degrading HMW-HA, which inhibits endothelial cell growth and angiogenesis in the cartilage region. Therefore, the lack of CEMIP is detrimental to normal osteogenesis (56). On the other hand, excessive CEMIP secretion is not conducive to the accumulation of HMW-HA, which reduces the viscosity of synovial fluid in the inflamed joint cavity and reduces the protective buffer effect (16). It has been confirmed in several studies that HMW-HA is anti-inflammatory and anti-angiogenic, and LMW-HA is pro-inflammatory and pro-angiogenic (81).

### 3.2 CEMIP promotes EGFR-associated phosphorylation levels

EGFR contains extracellular domain of ligand binding, hydrophobic region of single transmembrane and intracellular domain containing tyrosine-protein kinase activity, so it is also a class of receptor tyrosine kinases (RTKs) (82). EGF binds EGFR to form a dimer that activates the intracellular protein kinase pathway (83). This autophosphorylation can induce the phosphorylation of downstream signaling molecules (84). Previous studies have shown that the activation of EGFR signaling pathway is significantly related to the proliferation and invasion of various parenchymal tumor cells (85–89). CEMIP plays an important role in the process of EGF-dependent EGFR phosphorylation. The phosphorylation of one threonine residue site and three tyrosine residue sites in EGFR is decreased in cell lines with CEMIP deficiency (90, 91). Meanwhile, the phosphorylation levels of the major signaling pathways downstream of EGFR, mitogen-activated protein kinase kinase/ERK, PI3K/Akt, and STAT3 are seriously affected (11, 20, 35, 61, 63, 64, 91–93). Overexpression of CEMIP improves the phosphorylation level of EGFR and the above downstream signaling molecules, thus enhancing tumor cell metastasis (91).

### 3.3 CEMIP regulates the cellar microenvironment

The cellular microenvironment is the surrounding environment where cells exist, including non-parenchymal cells, such as immune cells like T and B cells, fibroblasts, vascular endothelial cells, and ECM, such as structural collagen, adhesion proteins, proteoglycans, and some soluble cytokines (94–98). All the above play a vital role in cellular activities. It has been reported that CEMIP not only acts on tumor cells to enhance their proliferation and metastasis ability but also contributes to cell survival and growth by modifying the structure or composition of the cell microenvironment (99, 100). Although the current research studies still have certain restrictions and cannot fully elucidate the function of CEMIP in the cellular microenvironment, it is worth affirming that the influence of CEMIP in the cellular microenvironment is of great significance to the survival and development of cells.

#### 3.3.1 CEMIP promotes tumor cell proliferation and metastasis

**Figure 4:**
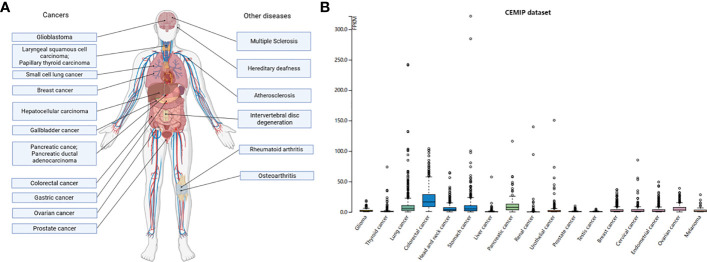
Expression levels of CEMIP in different diseases. **(A)** The expression of CEMIP in diseases. Human diseases characterized by the regulation of CEMIP. Considerable data now show that CEMIP is a contributing factor to the disease process and not simply a response to the condition. **(B)** RNA expression levels of CEMIP in different human cancers. RNA expression of CEMIP in different cancers from the Human Protein Atlas dataset.

In the process of tumorigenesis, we often use the TNM grading to judge the difficulty of tumor cure (101). The higher the grade, the worse the prognosis. The primary focus and distant metastases often determine whether the tumor is progressing in the direction of progression. In several studies, CEMIP has been confirmed as an oncogene, and the abnormal expression of CEMIP can be detected in most cancer cells, such as prostate cancer (PCa), small cell lung cancer (SCLC), breast cancer, hepatocellular carcinoma (HCC), GC, pancreatic cancer, colon cancer, pancreatic ductal adenocarcinoma (PDAC), and laryngeal squamous cell carcinoma (LSCC) (50, 102–115) (**Figure 4A**). In addition, the RNA expression in different cancers from the Human Protein Atlas Dataset (https://www.proteinatlas.org/) shows that the expression of CEMIP is highest in the colorectal cancer (**Figure 4B**). In gain- and loss-of-function experiments, the overexpression of CEMIP is accompanied by increased proliferation and migration of tumor cells, and CEMIP deficiency is associated with decreased in tumor cell proliferation and migration (51, 61, 105, 116–118).

Rodrigues et al. (119) constructed organoid models of mouse brains to elucidate the role of CEMIP from the three-dimensional organ level. Breast cancer cells were artificially planted on the model of brain organoid. Exosomes containing high concentrations of CEMIP were extracted as exogenous CEMIP additives. The exosomes with high concentrations of CEMIP and exosomes with low concentrations or even lack of CEMIP were added to the organoid models of the experimental group and the control group, respectively, and the number of breast cancer cells that colonized and invaded brain organoids in the experimental group was significantly higher than that in the control group. Moreover, when xenografting experiments were performed on mice, after adding exosomes with high concentrations of CEMIP, the proliferation of vascular endothelial cells in the brain was obvious, and vascular branches were significantly increased, which enhanced the colonization and invasion ability of breast cancer cells.

#### 3.32 CEMIP regulates immune cells

Once the tumor microenvironment is formed, numerous immune cells, such as T cells, myeloid-derived suppressor cells (MDSCs), macrophages, mast cells, granulocytes, and B cells, are chemotactic here, constituting the main stromal cells of the tumor microenvironment. The cells and molecules in the tumor microenvironment are in a dynamic process, reflecting the evolution of the tumor microenvironment. The end result is that a large number of immunosuppressive cells, such as MDSCs, regulatory T (Treg) cells, and tumor-associated macrophages (TAMs), and a large number of anti-inflammatory factors, such as IL-10 and TGF-β, are accumulated in the tumor microenvironment to jointly promote tumor immune escape, growth, and metastasis (48). CEMIP is also expressed in a variety of immune cells, especially in Treg cells, dendritic cells (DCs), naive B cells, memory B cells, and naive CD8^+^ T cells (117).

Zhang et al. (120) found that CEMIP specifically downregulated the expression of MHC-I on the surface of murine and human colon cancer cells, hindering the cytotoxicity of CD8^+^ T cells. In addition, they also demonstrated that the combination of CEMIP inhibition and immune checkpoint blockade (ICB) impeded tumor growth and enhanced therapeutic efficacy in colorectal cancer.

CEMIP has been shown to activate macrophages in the local tumor microenvironment (117, 121). Macrophages in the tumor microenvironment have the functions of drug resistance, promoting tumor angiogenesis and anti-tumor immunity. CEMIP plays an important role in guiding the penetration of tumor-associated macrophages. Studies show that the survival rate of glioma patients with high expression of CD163 (marker of macrophages) is significantly reduced (122, 123). The transplantation of human glioma cells knocked out of CEMIP into mice with CEMIP deficiency limited the proliferation and migration of tumor cells, and the penetration of macrophages in the glioma tissues of mice with CEMIP deficiency was decreased.

CEMIP also plays an important role in inducing macrophage polarization. The balance of M1/M2 macrophage polarization determines the fate of organs in inflammation or injury (124). M1 macrophages are pro-inflammatory cells and are polarized by lipopolysaccharide alone or in combination with Th1 cytokines [e.g., interferon gamma (IFN-γ) and granulocyte-macrophage colony-stimulating factor (GM-CSF)] and produce pro-inflammatory cytokines such as IL-1β, IL-6, IL-12, IL-23, and TNF-α (124). M2 macrophages have anti-inflammatory and immunomodulatory effects and are polarized by Th2 cytokines IL-4 and IL-13 to produce anti-inflammatory cytokines such as IL-10 and TGF-β (124). CEMIP was associated with M2 macrophages, and studies have shown a positive correlation between CEMIP and M2 macrophages infiltration (117, 125). Both infiltration and polarization of macrophages were attenuated in CEMIP knockout mice (122). It has been reported that the Wnt/β-catenin pathway enhances polarization of M2 macrophages, regulates the proliferation of tumor-associated macrophages, and promotes tumor progression (126, 127). CEMIP plays an important role in the Wnt/β-catenin signaling pathway (4). Therefore, CEMIP may activate the Wnt/β-catenin signaling pathway to stimulate the polarization of macrophages into M2 macrophages and to secrete corresponding anti-inflammatory factors, which may promote the immune escape of tumor cells and increase the probability of metastasis. In addition, CEMIP deficiency inhibits the expression of VEGF in macrophages (128).

#### 3.33 CEMIP and ECM

Tumor cell metastasis means the reconstruction of the ECM, the degradation of collagen in the surrounding tissues providing pathways for cancer cells to spread outward, and new angiogenesis offering nutrients for cancer cells (99, 119). Studies have clarified that overexpressing CEMIP exerts the role of hyaluronidase, which makes the solid tumors loose, contributing to cancer cells escaping from the *in situ* tissue and achieving distal diffusion.

Matrix metalloproteinases (MMPs) can degrade various proteins in the ECM, destroy the histological barrier to tumor cell invasion, and play a key role in tumor invasion and metastasis. It is considered to be the main proteolytic enzyme in tumor invasion and metastasis (129, 130). Three MMP family proteins (MMP2, MMP7, and MMP14) were reduced in CEMIP knockout cells, suggesting that CEMIP may stimulate MMP family enzyme activity and accelerate EMT in GC cells (68). In a study of osteosarcoma, IL-1β–mediated MMP-13 expression is inhibited when CEMIP is knocked out (12). Therefore, CEMIP may have a positive correlation with MMP2, MMP7, MMP13, and MMP14 and regulate tumor cell EMT through MMPs to promote tumor cell migration. Koike et al. (9) reported that ipriflavone can be a drug that inhibits the hyaluronidase activity of CEMIP and inhibits the expression of MMP1 and MMP3 in FLS cells stimulated by TNF-α. *In vivo* experiments confirmed that, after ipriflavone treatment, the levels of LMW-HA in the joint cavity and serum of mice were reduced. Therefore, we suspect that CEMIP may regulate the expression of MMP1 and MMP3. Further studies are needed to verify the relative relationship by which CEMIP regulates MMP1 and MMP3.

In the experiments on breast cancer brain metastasis, the overexpression of CEMIP promotes the proliferation of cerebral vascular epithelial cells. By injecting exosomes containing CEMIP, part of blood–brain barrier is destroyed in mice, which provides an opportunity for cancer cells to invade, and the high level of CEMIP can rebuild the vascular microenvironment. Endothelial cells and microglia show the activation of signaling pathways associated with inflammation and tumor invasion (119). Silencing of *CEMIP* notably inhibits angiogenesis *in vitro* and *in vivo* by increasing the expression of semaphorin 3A (**SEMA3A**) and by decreasing the expression of vascular endothelial growth factor A (VEGFA), vascular endothelial (VE)–cadherin, phosphorylated ephrin type-A receptor 2 (EphA2), and LMW-HA. Overexpression of CEMIP promotes angiogenesis by increasing secretory VEGFA. However, this activity can be reversed by the HA biosynthesis inhibitor 4-methylumbelliferone (4-MU) (131), which may be a potential tumor therapeutic target.

### 3.4 CEMIP suppresses apoptosis of tumor cells

Programmed cell death occurs after detachment from the ECM, which is called anoikis (132). Cancer cells have a certain chance to escape from anoikis after breaking away from the ECM and entering the blood, which is an important way for tumor cells to successfully metastasize (133). Zhang et al. (134) investigated that overexpressing CEMIP enhanced anoikis resistance in PCa cells *via* the adenosine monophosphate-activated protein kinase (AMPK)/glycogen synthase kinase 3beta (GSK3β)-catenin signaling pathway. circRNAs are important players in the occurrence and development of various malignancies. Circ_0004585 generated by the *CEMIP* gene plays a crucial role in resisting apoptosis in PCa cells. Circ_0004585 upregulates the expression of transmembrane 9 superfamily member 4 (TM9SF4) by combining with miR-1248, inhibiting the phosphorylation of mammalian target of rapamycin (mTOR) and promoting the inhibition of anoikis in PCa cells (33).

Semaphorin is a kind of neuron axon guiding factor and has been found to promote tumor apoptosis in tumor-related fields. Shostak et al. (26) proved that CEMIP protects CaSki cells (cervical cancer cells) from apoptosis induced by semaphorin. After continuously stimulated by semaphorin for 72 h, 14.40% of CEMIP-deficient cells were in the late stage of apoptosis, compared with only 1.68% of the control group. In addition, with the extension of time, the gap in apoptosis rate between the two groups was gradually increased.

Ferroptosis is another modality of apoptosis (135). Recently, it has indicated that upregulating CEMIP promotes ferroptosis resistance during ECM isolation by promoting the uptake of cystine in PCa cells. Meanwhile, silencing CEMIP deprives of its ability to promote cystine uptake and restrain ferroptosis. The interaction of CEMIP with inositol 1,4,5-triphosphate receptor type 3 (ITPR3) regulates Ca2^+^ leakage of the ER, activates calcium/calmodulin-dependent protein kinase II (CaMKII), and further promotes phosphorylation and nuclear localization of nuclear factor erythroid 2-related factor 2 (NRF2). This leads to the elevated transcription of solute carrier family 7 member 11 (SLC7A11) in PCa cells, finally improving the survival of tumor cells. The discovery of this pathway provides new insights into therapeutic strategies for metastatic PCa (136).

### 3.5 CEMIP regulates autoimmunity

High levels of CEMIP and CEMIP-induced degradation of LMW-HA have been detected in the synovial fluid, chondrocytes, or synovial membranes of diseased joints in patients with RA. Yang et al. (79) found that, compared with healthy people, there are remarkable upregulation of CEMIP expression and the increased proliferation and angiogenesis of synovium in patients with RA. Similarly, Zhang et al. (13) demonstrated that CEMIP is secreted in serum and that synovial fluid secretion is positively correlated with LMW-HA in patients with RA, and the positive correlation represents a more obvious trend as the disease gets worse.

In multiple sclerosis (MS), HA degradation lesions and CEMIP expression are colocalized. Marella et al. (137) constructed an experimental autoimmune encephakmyelitis (EAE) animal model, observed a high expression of CEMIP in focal demyelinating lesions, and found a large aggregation of degraded HA at the lesion tissue. They found that CEMIP is expressed primarily by activated astrocytes invading damaged tissue. Similar results were observed in the tissues of a patient with MS. Inhibition of CEMIP expression in astrocytes may become a novel therapeutic strategy for MS.

### 3.6 CEMIP and infection

*Staphylococcus aureus* (*S. aureus*) and group A *streptococcus* (GAS) are the main bacterial pathogens that cause invasive infections of human skin. They have different means of invasion (138). GAS mimics HA-rich ECM in the environment around the dermis layer by expressing long-chain HA on its surface, thus preventing being killed by leukocytes (139). *S. aureus* also adapts to HA and uses its hyaluronidase to strengthen virulence (140).

Dokoshi et al. (70) chose *S. aureus* as a model to explore the interaction between the bacteria and the skin. After normal mice were infected by *S. aureus*, the *CEMIP* mRNA level of wound skin was increased. In the mice with knockout of the *CEMIP* gene, the proliferation and infection abilities of *S. aureus* were significantly reduced, and the skin wound recovered faster than that in normal mice (70). LMW-HA produced by the degradation of CEMIP provided numerous signals of injury. The infiltration of local neutrophils, DCs, and monocytes was increased in *CEMIP^−/−^* mice, which may help fight against infection. However, the expression of T-box transcription factor (Tbet)^+^, retinoid-related orphan receptor gamma T (RORgt)^+^, IFN-γ^+^, and IL-17^+^T in the spleen was decreased. It is suggested that the lack of CEMIP expression may increase the local inflammatory reaction but reduce the systemic inflammatory reaction after *S. aureus* infection, which means that the systemic response of *S. aureus* infection is reduced.

### 3.7 Promotion of fibrosis

Fibrosis is not a kind of disease, but the result of tissue repair after damage (141). When the local tissue is damaged, fibroblasts are activated and contracted, secreting inflammatory cytokines and ECM components, such as collagen and fibronectin (142). However, when the injury is repeated or serious, the continuous accumulation of ECM components will lead to tissue structure disorder, organ dysfunction, and eventually organ failure (143).

Studies have shown that CEMIP can activate fibroblasts and induce fibrosis. Deroyer et al. (19) found that CEMIP in OA cartilage from human and mouse was significantly higher than that in the healthy group, and the fibrosis-related indicators, COL1A1, COL3A1, and α-SMA, were significantly increased to aggravate the degree of joint fibrosis. Further research studies showed that CEMIP regulates a process of pro-fibrosis through the TGF-β signaling pathway, suggesting that CEMIP is probably an inducer of transdifferentiation of chondrocytes into “chondro-myo-fibroblasts.” In their subsequent study, CEMIP also showed a similar role of pro-fibrosis in the synovial membrane (7).

During lung injury, excessive activation and proliferation of fibroblasts can cause pulmonary fibrosis (144). Pirfenidone is a drug for the treatment of idiopathic pulmonary fibrosis (IPF); however, it is not clear whether its diagnosis target is lung fibroblasts (145). In the study of Kwapiszewska et al. (69), CEMIP was considered to be involved in the treatment of IPF with pirfenidone. The CEMIP level of patients with IPF was higher than that of the healthy group. After 7 months of pirfenidone treatment, the level of pulmonary fibrosis was decreased, and the level of CEMIP was also declined significantly. Subsequently, CEMIP gene silencing was performed on fibroblasts of IPF, which reduced the amount of collagen produced by fibroblasts and weakened the ability of cell proliferation and migration.

## 4 Potential applications of CEMIP

### 4.1 CEMIP was used as a therapeutic target

In view of the numerous and meaningful physiological and pathological functions shown by CEMIP, it is promising to design drugs targeting CEMIP to alleviate OA, anti-infection, delay skin aging, and combat tumor proliferation and migration (**Table 2**).

**Table 2:** Related applications of CEMIP as a therapeutic target.

Transfer method	The molecules contained	Therapeutic target	Mechanism	Reference
NA	Anti-CEMIP monoclonal antibodies	The joints of mice with OA	Antibody neutralizes CEMIP	(13)
NA	PTX	Colon cancer cells	Stabilizing microtubules inhibited the promigratory effect of CEMIP	(10)
Core–shell drug depot	PTX and CEMIP-specific shRNA	MDA-MB-231	Tumor-specific penetration, targeted drugs release	(42)
Thermosensitive PLGA-PEG-PLGA polymer	Dox and CEMIP-specific shRNA	Mouse breast carcinoma 4 T1 cells	Tumor-specific penetration, targeted and quantitative drug release	(43)

NA, not applicable.

#### 4.1.1 Therapeutic strategy of OA

One of the damaging consequences of OA is the degradation of HA, which leads to a reduction in relative molecular weight, concentration, and active components. This reduces the mechanical characteristics of the joint cavity and causes joint dysfunction. Therefore, intra-articular injection of exogenous HA can restore the concentration of HA in synovial fluid, help increase the lubrication effect of synovial fluid, and achieve the purpose of alleviating joint dysfunction (146). Another symptom is the release of inflammatory cytokines, which prompt immune cells to attack the injured area. In addition, nonsteroidal anti-inflammatory medicines that block the action of inflammatory cytokines are another method for treating arthritis (147).

In the previous chapters, we mentioned that the expression and activity of CEMIP have increased in inflammatory cytokine-mediated OA, which further induces more inflammatory cytokines expression. In addition, excessive CEMIP accelerates the degradation of HMW-HA into LMW-HA in joint cavity, breaking the balance between synthesis and degradation of HA. Zhang et al. (13) injected an anti-CEMIP monoclonal antibody into the joints of OA mice, effectively alleviating the severity of arthritis in mice and reducing the serum LMW-HA level and cytokine secretion. Koike et al. (9) treated mouse chondrosarcoma cells with ipriflavone and found that the expression of CEMIP decreased. *In vivo* experiments, ipriflavone was found to increase the accumulation of HA and inhibit the expression of MMP1 and MMP3, effectively relieving the disease.

Therefore, inhibiting the expression and activity of CEMIP can restore the balance between HA synthesis and degradation while simultaneously reducing the secretion level of joint inflammatory factors, which may be a new strategy for the treatment of OA.

#### 4.1.2 Therapeutic strategy of bacterial infection

Skin wound bacterial infection frequently results in inflammatory reactions, extensive ulceration, and even sepsis, a potentially fatal condition. Moreover, with the increase of drug-resistant bacteria, treatment is becoming more and more difficult, resulting in prolonged wound healing. Conventional treatments include debridement surgery, antibiotics, and wound dressings (148). Dokoshi et al. (70) found that, after *S. aureus* infection in mice, the expression of CEMIP was elevated and the process of bacterial invasion into the skin was accelerated by degrading HA. However, it remains unclear how bacterial invasion induces CEMIP production, why only CEMIP was elevated, and whether this phenomenon occurs across different bacterial infections. Understanding how the skin initiates the digestion of HA has important diagnostic and therapeutic implications for many infectious and inflammatory diseases. Perhaps adding CEMIP-related inhibitors to the currently used dressing can enhance its efficacy, which is also something that we need to explore.

#### 4.1.3 Delay skin aging

The skin has various biological purposes as a body organ that is continuously in contact with the outside world. With age and environmental stresses, such as ultraviolet radiation, damage from pollutants and microorganisms can accelerate skin damage (149). A network structure that is composed of HA and proteoglycans envelops collagen and elastin fibers to maintain skin elasticity (149). Research by Yoshida et al. (54) indicated that CEMIP expression was increased in photoaged skin compared with that in normal skin, and the expression of HAS1/2 was decreased. Meanwhile, the expression of CEMIP was negatively correlated with the amount of HA in the dermal papilla of photoaged skin. CEMIP-mediated degradation of HA may lead to a decrease in the molecular weight and quantity of HA in the dermal papillae of photoaged skin. As a result, it destroys the integrity of the dermal ECM and shows the symptoms of photoaging, such as skin wrinkles. Therefore, downregulating CEMIP expression or inhibiting CEMIP-mediated HA degradation in dermal cortex may provide an effective new therapy for preventing photoaged skin damage.

#### 4.1.4 Tumor-related treatment strategies

In previous chapters, we have outlined the existing information on the role of CEMIP in tumor cell proliferation and metastasis. Our current understanding of the molecular mechanisms involved in CEMIP is inadequate, but its potential applications are worth exploring.

Studies have demonstrated that targeted delivery of shCEMIP prevents CEMIP expression and is crucial for preventing tumor cell proliferation and migration (60). This work provided additional evidence that suppressing CEMIP expression can prevent the growth of tumors, and it suggested that future research might focus on developing CEMIP inhibitors. In a study on colorectal cancer (10), CEMIP increased the phosphorylation of PP2A. Through the influence of PP2A on microtubule instability, stathmin, the downstream component of the CEMIP-PP2A complex, increased cell motility. PTX, as a drug that can stabilize microtubules and inhibit the effect of CEMIP, thus, attenuated the movement of colon cancer cells. Whether other similar microtubule stabilizers, such as docetaxel, ebomycin, and discodermolide6, have similar functions needs further investigation.

#### 4.1.5 Serum CEMIP as a diagnostic marker

CEMIP, as a secreted protein, has been poorly studied as a serum biomarker. Research has shown that the combined detection of CEMIP and CA19-9 improves the diagnostic accuracy of pancreatic cancer (105). Higher CEMIP expression was also detected in blood samples from patients with metastatic liver cancer compared with that from healthy individuals, inconsistent with previous studies that reported lower CEMIP expression in liver tissue. In OA and RA, serum CEMIP expression also increased to varying degrees (12, 13). The current studies are all based on small sample size, lacking the support of large sample size, and the relevant diagnostic specificity and sensitivity need to be further studied.

### 4.2 Drug delivery system

For metastatic tumors, because of the lack of a deep understanding of the mechanism and effective drug delivery methods, the current treatment methods are difficult to defeat metastatic tumors. Reprogramming of intelligent nanodrug delivery system using tumor microenvironment has become a hot topic in combined anti-tumor therapy.

Dong et al. (42) proposed a core–shell drug depot consisting of a micellar core and a cross-linked gel shell for the site-directed shuttle of PTX and CEMIP-specific shRNA. Poly (E-caprolactone) was grafted onto a polyethyleneimine branched surface (PEI-PCL), and hydrophobic PTX was embedded into the micellar core. CEMIP and PEI were condensed by electrostatic interaction. Then, HA was coated as a shell. The nanoscale drug depot has a hyaluronidase (HAase)-triggered charge conversion and a desirable release profile. Upon arrival at the designated region, the HA shell is degraded by concentrated HAase, facilitating drug transport to individual subcellular targeting sites. The rapid transport of micellar core within the cell achieves endolysosome escape and cytoplasmic liberation. Moreover, the interference with CEMIP expression by sustained RNA interference (RNAi) can affect a variety of functions such as apoptosis, migration, and invasion.

Meanwhile, Wang et al. (43) proposed a similar method. Specifically, a thermosensitive Poly(D,L-lactide-co-glycolide)-b-poly(ethyleneoxide)-b-poly(D,L-lactide-co-glycolide) polymer was introduced as an injectable hydrogel matrix, whose dosage volume and frequency were carefully controlled according to tumor size and gel degradation kinetics. Structurally, doxorubicin (Dox) and arginine-terminated nanoparticles containing CEMIP-specific shRNA were incorporated into the hydrogel, resulting in a local and sustained drug reservoir for synergistic therapy. In addition, Dox was used to block DNA replication/transcription, and shCEMIP was used to continuously silence CEMIP expression to regulate the invasive phenotype. It showed good results after the animal experiments.

In general, the high expression of CEMIP in metastatic tumors can be used to design drug nanoparticles coated with HA. When the nanoparticles arrive in the tumor environment with a high level of CEMIP in the circulatory system, HA can be degraded by CEMIP secreted from the tumor, and anti-tumor drugs wrapped in the core are released to achieve the purpose of precise drug delivery. However, the difficulty of implementing this scheme lies in precisely controlling the thickness of HA shell or adding new materials to prevent the free hyaluronidase in the circulatory system from degrading HA shell in advance. It can be predicted that this scheme has a huge application prospect.

## 5 Conclusions

Overall, our understanding of CEMIP has made great progress in the 20 years since it was first discovered. The main contribution of this review is that we summarized the structure of CEMIP, the factors that regulate CEMIP expression, the molecular mechanisms of CEMIP, and its potential clinical applications. In addition to the role of HA degradation, CEMIP plays an important role in regulating the internal physiological activities of cells, such as activating phosphorylation, regulating cell microenvironment, inhibiting cell apoptosis and promoting tumor cell activity, and changing the external environment to promote cell growth. This review provides a comprehensive and detailed understanding for those who want to understand CEMIP.

However, many challenges remain. First is whether CEMIP plays some important and unknown role in some pathophysiological processes of non-tumor diseases. For example, what is the specific mechanism by which inflammatory factors regulate CEMIP, and how does the high concentration of CEMIP further aggravate the inflammatory process? Second is whether such a positive feedback mode of regulation really exists or whether there are some new molecular mechanisms in this process that are unknown. Furthermore, CEMIP has been shown to promote EGFR pathway-related phosphorylation (26). The regulatory mechanism of CEMIP in these protein post-translational modifications (PTMs) remains unknown. Third is whether CEMIP also promotes PTMs in other ways, such as glycosylation, ubiquitination, and methylation to regulate the expression of some proteins or not (150). It also requires further work to understand the molecular function of the CEMIP protein in PTMs.

Although our understanding of CEMIP is still limited, the characteristics observed from studies suggest that it can be used as a serum biomarker to predict the development of disease (105, 151), but, for now, we need more clinical sample data to further verify its sensitivity and specificity. Meanwhile, combined with the drug delivery technology described above, CEMIP can accurately treat tumors, inflammation, and other diseases. A new generation of wound dressings may be created as a result of research into the unique mechanism of CEMIP in the immunological process of skin wound infection. However, further research is needed on how to develop these applications.

## Author contributions

YL and GH drafted the manuscript. YTL, XYK, KMY, and ZLL collected the related references and illustrated figures. WWL, JXL, JHZ, and STZ collected the related references and illustrated tables. YXL, CLB, and AXZ conceived the project and edited the manuscript. All authors read and approved the final manuscript.

## Glossary

**Table:**

CEMIP	cell migration–inducing protein
HYBID	hyaluronan-binding protein involved in hyaluronan depolymerization
HA	hyaluronic acid
ECM	extracellular matrix
ER	endoplasmic reticulum
HSPA5	heat shock protein A5
BiP	binding immunoglobulin protein
PCa	prostate cancer
SCLC	small cell lung cancer
HCC	hepatocellular carcinoma
GC	gastric cancer
PDAC	pancreatic ductal adenocarcinoma
LSCC	laryngeal squamous cell carcinoma
IL-6	interleukin-6
LMW-HA	low–molecular weight hyaluronan
DAMP	damage-associated molecular pattern
OA	osteoarthritis
HMW-HA	high–molecular weight hyaluronan
RA	rheumatoid arthritis
FLS	fibroblast-like synoviocyte
TNF-α	tumor necrosis factor–alpha
TGF-β1	transforming growth factor–beta 1
ATF3	activating transcription factor 3
Bcl-2	B-cell lymphoma-2
REST	repressor element 1 silencing transcription factor
RE1	repressor element 1
ncRNAs	non-coding RNAs
miRNAs	micro RNAs
circRNAs	circle RNAs
lncRNAs	long non-coding RNAs
ceRNAs	competing endogenous RNAs
MSI 1	Musashi RNA-binding protein 1
H3K27me3	trimethylation of lysine on histone H3
TNBC	triple-negative breast cancer
HIF-2α	hypoxia-inducible factor 2α
RTKs	receptor tyrosine kinases
MDSCs	myeloid-derived suppressor cells
Treg	regulatory T
TAM	tumor-associated macrophage
MMP	matrix metalloproteinase
DCs	dendritic cells
VEGF	vascular endothelial-derived growth factor
ICB	immune checkpoint blockade
SEMA3A	semaphorin 3A
VEGFA	vascular endothelial growth factor A
VE	vascular endothelial
EphA2	ephrin type-A receptor 2
4-MU	4-methylumbelliferone
TM9SF4	transmembrane 9 superfamily member 4
ITPR3	inositol 1,4,5-triphosphate receptor type 3
CaMKII	calcium/calmodulin-dependent protein kinase II
NRF2	nuclear factor erythroid 2-related factor 2
SLC7A11	solute carrier family 7 member 11
EAE	encephakmyelitis
MS	multiple sclerosis
*S. aureus*	*Staphylococcus aureus*
GAS	group A *streptococcus*
Tbet	T-box transcription factor
RORgt	retinoid-related orphan receptor gamma T
IFN-γ	interferon gamma
IPF	idiopathic pulmonary fibrosis
PTX	paclitaxel
Dox	doxorubicin
EGFR	epidermal growth factor receptor
STAT3	signal transducer and activator of transcription 3
PTMs	post-translational modifications.
